# Telehealth‐aided outpatient management of acute heart failure in a specialist virtual ward compared with standard care

**DOI:** 10.1002/ehf2.15003

**Published:** 2024-08-13

**Authors:** Rajiv Sankaranarayanan, Debar Rasoul, Naomi Murphy, AnneMarie Kelly, Siji Nyjo, Carolyn Jackson, Jane O'Connor, Peter Almond, Nisha Jose, Jenni West, Rosie Kaur, Chukwemeka Oguguo, Homeyra Douglas, Gregory Y.H. Lip

**Affiliations:** ^1^ Liverpool University Hospitals NHS Foundation Trust, Aintree Hospital Liverpool UK; ^2^ Liverpool Centre for Cardiovascular Science at University of Liverpool, Liverpool John Moores University and Liverpool Heart & Chest Hospital Liverpool UK; ^3^ GIRFT (Getting It Right First Time) NHS England London UK; ^4^ North West Coast Cardiac Clinical Network NHS England London UK; ^5^ Mersey Care NHS Foundation Trust Liverpool UK; ^6^ Health Technology and Access Services, Community Services Division Mersey Care NHS Foundation Trust Liverpool UK; ^7^ Health Innovation North West Coast Academic Health Sciences Network Liverpool UK; ^8^ CCIO Medical Lead for Remote Monitoring Cheshire and Merseyside Mersey Care NHS Foundation Trust Liverpool UK; ^9^ Danish Center for Health Services Research, Department of Clinical Medicine, Aalborg University Aalborg Denmark

**Keywords:** Acute heart failure, Hospitalization, Mortality, Telehealth, Virtual ward

## Abstract

**Aims:**

The aim of this propensity score matched cohort study was to assess the outcomes of telehealth‐guided outpatient management of acute heart failure (HF) in our virtual ward (HFVW) compared with hospitalized acute HF patients.

**Methods and results:**

This cohort study (May 2022–October 2023) assessed outcomes of telehealth‐guided outpatient acute HF management using bolus intravenous furosemide in a HF‐specialist VW. Propensity score matching (PSM) was performed using logistic regression to adjust for potential differences in baseline patient characteristics between HFVW and standard care [Get With The Guidelines‐HF score, clinical frailty score (CFS), Charlson co‐morbidity index (CCI), NT‐proBNP, and ejection fraction]. Clinical outcomes (re‐hospitalizations and mortality) were compared at 1, 3, 6, and 12 months versus standard care‐SC (acute HF patients managed without telehealth in 2021). Five hundred fifty‐four HFVW ADHF patients (age 73.1 ± 10.9 years; 46% female) were compared with 404 ADHF patients (74.2 ± 11.8; *P* = 0.15 and 49% female) in the standard care‐SC cohort. After propensity score matching for baseline patient characteristics, re‐hospitalizations were significantly lower in the HFVW compared with SC (1 month‐HFVW 8.6% vs. SC‐21.5%, *P* < 0.001; 3 months‐21% vs. 30%, *P* = 0.003; 6 months‐28% vs 41%, *P* < 0.001 and 12 months‐47% vs. 57%, *P* = 0.005) and mortality was also lower at 1 month (5% vs. 13.7%; *P* < 0.001), 3 months (9.5% vs. 15%; *P* = 0.001), 6 months (15% vs. 21%; *P* = 0.03), and 12 months (20% vs. 26%; *P* = 0.04). Multivariate logistic regression analysis showed that compared with standard care, HFVW management was associated with lower odds of readmission (1‐month odds ratio (OR) = 0.3 [95% Confidence Interval CI 0.2–0.5], *P* < 0.0001; 3 month OR = 0.15 [0.1–0.3], *P* < 0.0001; 6‐month OR = 0.35 [0.2–0.6], *P* = 0.0002; 12‐month OR = 0.25 [0.15–0.4], *P* ≤ 0.001 and mortality (1‐month OR = 0.26 [0.14–0.48], *P* < 0.0001; 3‐month OR = 0.11 [0.04–0.27], *P* < 0.0001; 6‐month OR = 0.35, [0.2; 0.61], *P* = 0.0002; 12‐month OR = 0.6 [0.48; 0.73], *P* = 0.03. Higher GWTG‐HF score independently predicted increased odds of re‐hospitalization (1‐month OR = 1.2 [1.1–1.3], *P* < 0.001; 3‐month OR = 1.5 [1.37; 1.64], *P* < 0.0001; 6‐month OR = 1.3 [1.2–1.4], *P* < 0.0001; 12‐month OR = 1.1 [1.05–1.2], *P* = 0.03) as well as mortality (1‐month OR = 1.21 [1.1–1.3], *P* < 0.0001; 3‐month OR = 1.3 [1.2–1.4], *P* < 0.0001; 6‐month OR = 1.2 [1.1–1.3], *P* < 0.0001; 12‐month OR = 1.3 [1.1–1.7], *P* = 0.02). Similarly higher CFS also independently predicted increased odds of re‐hospitalizations (1‐month OR = 1.9 [1.5–2.4], *P* < 0.0001; 3‐month OR = 1.8 [1.3–2.4], *P* = 0.0003; 6‐month OR = 1.4 [1.1–1.8], *P* = 0.015; 12‐month OR 1.9 [1.2–3], *P* = 0.01]) and mortality (1‐month OR = 2.1 [1.6–2.8], *P* < 0.0001; 3‐month OR = 1.8 [1.2–2.6], *P* = 0.006; 6‐month OR = 2.34 [1.51–5.6], *P* = 0.0001; 12‐month OR = 2.6 [1.6–7], *P* = 0.02). Increased daily step count while on HFVW independently predicted reduced odds of re‐hospitalizations (1‐month OR = 0.85[0.7–0.9], *P* = 0.005), 3‐month OR = 0.95 [0.93–0.98], *P* = 0.003 and 1‐month mortality (OR = 0.85 [0.7–0.95], *P* = 0.01), whereas CCI predicted adverse 12‐month outcomes (OR = 1.2 [1.1–1.4], *P* = 0.03).

**Conclusions:**

Telehealth‐guided specialist HFVW management for ADHF may offer a safe and efficacious alternative to hospitalization in suitable patients. Daily step count in HFVW can help predict risk of short‐term adverse clinical outcomes.

## Introduction

Heart failure (HF) affects nearly 65 million people worldwide and has been described to be a global pandemic.^1^ The need for hospitalization is a crucial inflection point in the lifetime of a patient with HF and is typically due to sudden worsening of symptoms due to fluid overload. This condition is termed acutely decompensated heart failure (ADHF) and usually necessitates intravenous (IV) diuretics. The number of hospitalizations has been shown to be an independent predictor of poor quality of life as well as increased mortality.^2^, ^3^, ^4^, ^5^ There are nearly 100 000 hospitalizations per year with a primary diagnosis of HF in the UK.^6^ In addition to the significant adverse impact on patient outcomes, hospitalizations also pose a burden on healthcare systems due to the financial impact caused^7^ and resource implications due to hospital bed‐shortages.^8^ Additionally, hospitalization can also be associated with complications such as hospital‐acquired infections,^9^ delirium,^9^ falls and poor nutrition, which in turn lead to worse outcomes.^9^ There has therefore been an increasing focus on outpatient management of ADHF.

Our local experience of telehealth monitoring for long‐term health conditions (LTC), such as heart failure, chronic obstructive pulmonary disease (COPD), and diabetes mellitus, has shown a net reduction in annual emergency admissions of 22.7%^10^ in a large retrospective observational study with matched anonymous controls spanning 4 years and over 5000 patients. These findings are in line with similar studies of large patient groups.^11^, ^12^, ^13^, ^14^

The concept of ‘virtual ward’ (VW) for ADHF builds on the experience gained from managing other acute conditions such as COVID,^15^ acute respiratory infections, and frailty syndromes in the home setting with the aid of telehealth remote monitoring technology.^16^ A virtual ward offers an alternative to hospitalization or early supported discharge (ESD) for suitable patients with ADHF, by offering treatment and monitoring by HF specialist teams with the aid of remote health technology in their usual place of residence. While outcomes have been described from the use of VW models for other conditions, there is a paucity of evidence for VW management of ADHF. The aim of this propensity score matched cohort study was to assess the outcomes of our acute HFVW in comparison to a cohort of hospitalized ADHF patients.

## Methods

**Figure 1:**
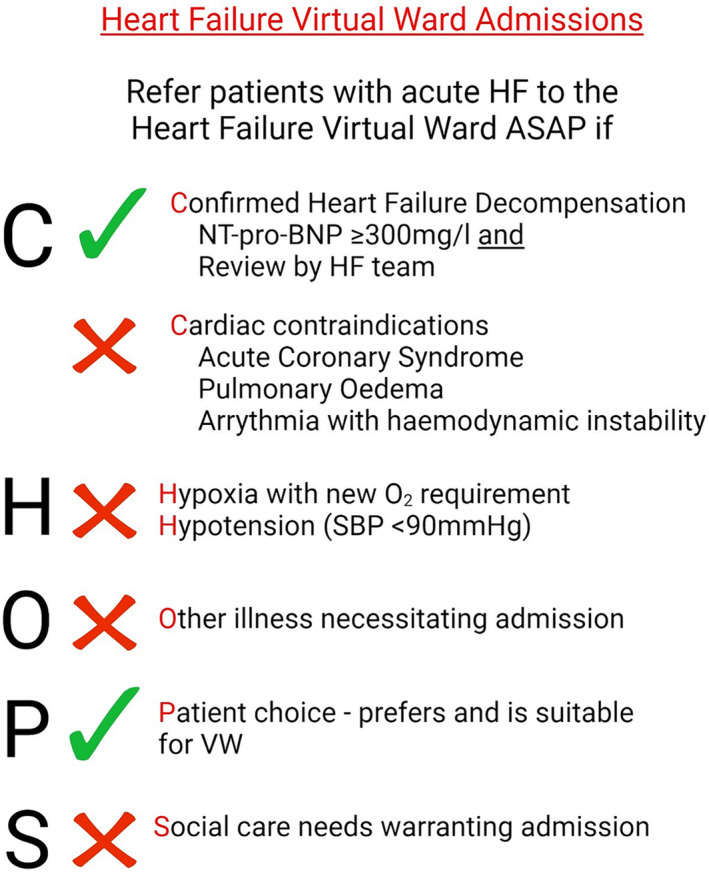
Inclusion and exclusion criteria for HFVW.

This was a retrospective analysis of a prospective cohort study of consecutive patients with acute heart failure managed in the acute HFVW at Liverpool University Hospitals NHS Foundation Trust (Aintree University Hospital and Royal Liverpool University Hospital) from 16 May 2022 to 31 October 2023. This cohort was compared with a ‘standard care (SC)’ cohort in 2021, consisting of patients who were hospitalized for acute heart failure (when telehealth remote monitoring was not available to aid out of hospital management of acute HF). Set‐up of our HFVW was enabled through a Digital Transformation award from NHS Transformation Directorate (previously NHSX). Adult patients with acute HF (new diagnosis or acute worsening in those with a known previous diagnosis of HF) were included based on inclusion and exclusion criteria (shown in *Figure* *1*) if the patient or their carer could provide consent to be managed in the HFVW as an alternative to hospitalization or for early supported discharge. The study complies with the Declaration of Helsinki, the institutional Research and Innovation department committee has approved the study protocol and informed consent was obtained from the subjects (or their guardians).

**Figure 2:**
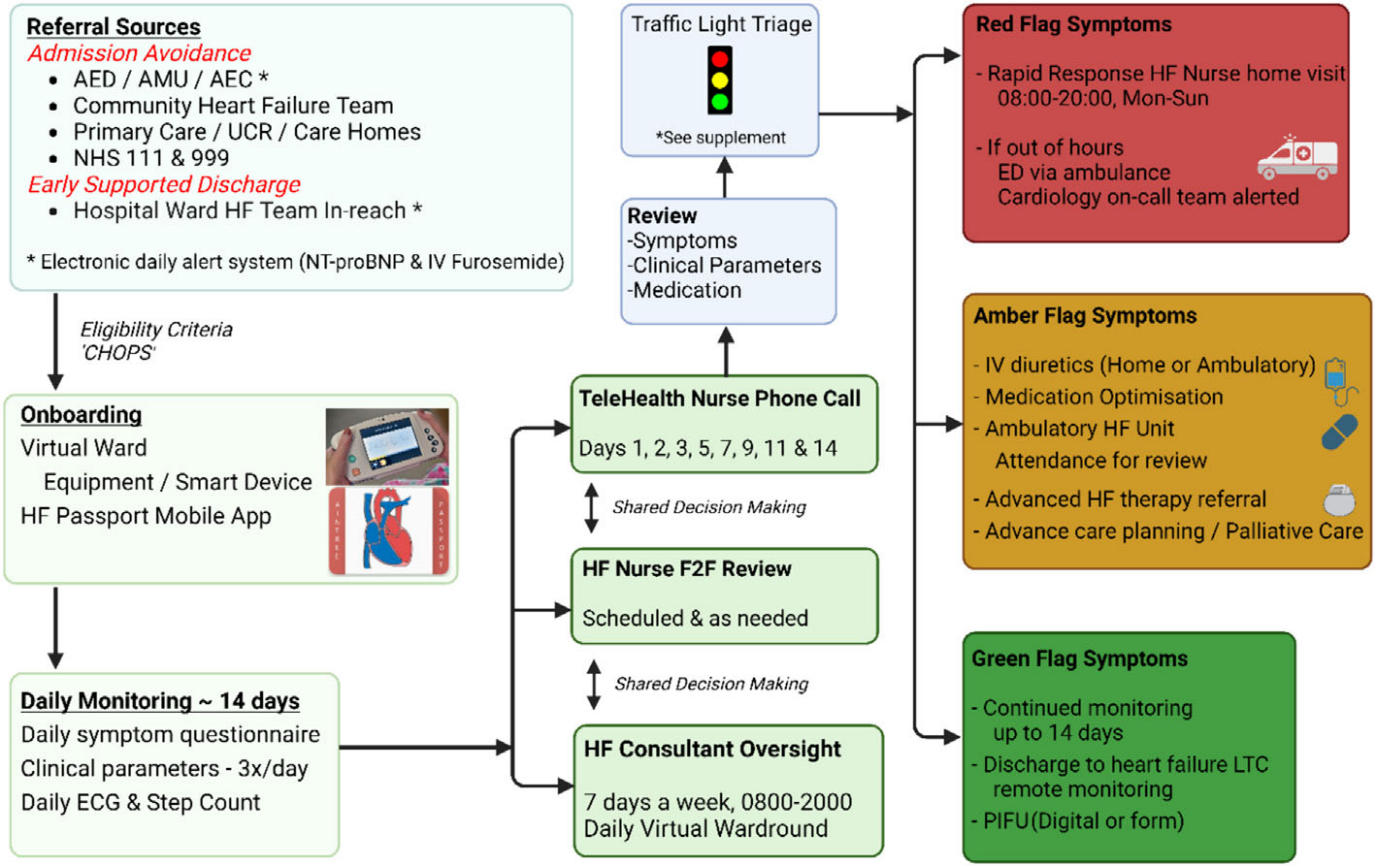
HFVW clinical pathway.

The HFVW referrals consisted of (1) ‘Admission Avoidance’ ‐ sources of referral included accident and emergency department (AED) and ambulatory units of three large university teaching hospitals in Liverpool ‐ Aintree University Hospital and Royal Liverpool University Hospital (Liverpool University Hospitals NHS Foundation Trust) and Liverpool Heart and Chest Hospital, outpatient clinics, community HF specialist nurses from teams in and around Liverpool (Merseycare NHS Foundation Trust, Liverpool Community Heart Failure team, Knowsley community heart failure team, West Lancashire Community heart failure team), primary care and (2) ‘Early Supported Discharge’ (hospital wards from three hospitals in Liverpool). There was a dedicated HF specialist nurse (‘case‐finder’) available from 8 am to 8 pm 7 days a week to receive referrals and assess patient suitability for HFVW. The Liverpool HFVW patient pathway was created following a series of meetings conducted (January to March 2022) including a multidisciplinary team including HF specialist consultants, HF specialist nurses, HF specialist pharmacist, telehealth specialist nurses, information technology specialists, and outcomes research analysts from the partner organizations mentioned above, University of Liverpool and Health Innovation North‐West Coast. Suitable patients were ‘onboarded’ onto the HFVW remote monitoring digital health platform (DOC@HOME®) and equipment delivered for daily monitoring [weighing scales for daily weights, BP, pulse, oxygen saturations thrice a day, single lead electrocardiogram (ECG) and a wearable for step count]. Patients also received a daily symptom questionnaire (*Figure* *2*). Patients received daily phone calls from telehealth nurses, face‐to‐face clinical assessment when on ambulatory or home intravenous diuretics and as and when required, through integration of hospital and community HF specialist nursing teams. Patients with deteriorating clinical symptoms or signs who required a prompt clinical assessment could be reviewed at home by a dedicated HF specialist nurse (available between 8 am and 8 pm 7 days a week – ‘rapid response HF specialist nurse’). HFVW patients were also provided with access to mobile phone application (Aintree Heart Failure Passport) to improve patient education, self‐care behaviour and prompts for medication adherence, appointments. There was a daily HFVW round conducted by a heart failure consultant cardiologist 7 days a week with clinical cover provided from 8 am to 8 pm, seven days a week. Patients with ‘red‐flag symptoms’ out of HFVW working hours (8 pm–8 am) were advised to contact paramedics and were supported for clinical advice by the hospital on‐call cardiology team. Patient received intravenous furosemide administered as a bolus (no faster than 4 mg/min) using an elastomeric pump (Accufuser®) in their home (if Rockwood Clinical Frailty Score was >6) or in a HF specialist delivered ambulatory HF unit. We also used point of care blood tests (iSTAT) for renal function testing and point of care echocardiography using the Vscan™. Remote electronic prescribing was enabled to allow rapid initiation and optimization of prognostic HF therapies, and a HF specialist pharmacist also conducted HFVW medication optimization clinics. Patients with heart failure with reduced ejection fraction (HFrEF) or heart failure with mildly reduced ejection fraction (HFmrEF) and iron deficiency also received out‐patient intravenous iron replacement as per ESC guidelines.^17^ At the time of discharge from HFVW, patients were stepped down to the long‐term conditions remote monitoring segment where they could be monitored for up to 6 months. Patients from HFVW could also be referred to the virtual multi‐specialty multidisciplinary team (consisting of primary care, secondary and tertiary care HF consultants, hospital and community HF specialist nurses, specialists in diabetes, nephrologist, geriatrician, palliative care, chest physician, pharmacologist, and pharmacist) for improved management of co‐morbidities in HF patients.^18^ The HFVW care pathway flow chart is also illustrated in *Figure* *2*.

Baseline patient characteristics were collected from the electronic patient record, such as age, gender, clinical parameters at the time of presentation, routine blood tests, ECG, and echocardiogram results. We calculated the Charlson co‐morbidity index (CCI)^19^ by computing details regarding multimorbidity. We also assessed frailty using the Rockwood Clinical Frailty Scale (CFS).^20^ Clinical risk was estimated using the Get with the Guidelines (GWTG) Heart Failure risk score (calculated from data at the time of first presentation) and using the National Early Warning Score (NEWS2)^21^ on a daily basis. GWTG‐HF risk score^22^ is a validated HF risk score for in‐hospital mortality and includes systolic blood pressure, serum sodium blood urea nitrogen (BUN), age, heart rate, history of COPD and race (detailed in supplementary information). HF therapies were compared between the two cohorts, including prognostic quadruple HFrEF therapy (beta‐blocker, angiotensin converting enzyme inhibitor [ACEi], angiotensin receptor blocker [ARB], angiotensin receptor blocker‐neprilysin inhibitor [ARNI], mineralocorticoid receptor antagonist [MRA], and sodium‐glucose cotransporter 2 inhibitors [SGLT‐2i]), implant of cardiac resynchronization therapy pacemaker (CRTp), implantable cardiovertor‐defibrillator (ICD), referral for cardiac transplant and advance care planning discussions including referral to palliative care. We collected clinical outcome measures such as rehospitalization (all‐cause as well as HF) and mortality, which were assessed at 1 month, 3 months, 6 months, and 12 months. We assessed the patient's step‐count daily using a wearable monitor and calculated the percentage change in step count on the day of discharge from the HFVW compared with that on the day of admission to HFVW. We also compared the incidence of hospital acquired infection, falls and adverse drug reactions between the two cohorts. In addition to GWTG, CCI, and CFS, we included additional variables (not used for GWTG or CCI) in multivariate logistic regression analysis such as sex, baseline NYHA class, change in step count, NT‐proBNP, and left ventricular ejection fraction (LVEF).

### Inclusion and exclusion criteria

Adult patients (aged ≥18 years) with a confirmed diagnosis of acute HF (new or known diagnosis‐ESC criteria) were included in the study. Patients recruited to the HFVW were consented (if the patient was unable to provide consent due to cognitive problems, their next of kin or carer provided consent on their behalf, and the carer also assisted the patient in providing daily telehealth readings). Exclusion criteria included patients with ongoing acute coronary syndrome, pulmonary oedema (based on clinical findings of bilateral lung crepitations accompanied by typical radiological signs on chest X‐ray), evidence of haemodynamic instability (systolic blood pressure <90 mmHg, unless known to be usual for patient), heart rate ≥130 b.p.m. or ≤40 b.p.m., untreated malignant arrhythmias (ventricular arrhythmias and higher degree AV block), new oxygen requirement, other medical, or surgical conditions that would require hospitalization and complex social needs.

We employed the use of an acronym ‘CHOPS’ criteria (shown in *Figure* *1*) as an aide memoire to enable referrers to remember and easily understand inclusion and exclusion criteria.

### Statistical analysis

For the descriptive statistics of our patient population, we represented continuous variables as means with standard deviations (mean ± SD) if normally distributed and compared using the Student's *t*‐test. For non‐normal data, the parameters were described using medians with interquartile ranges and compared using the Mann–Whitney test. Categorical data were expressed as percentages and compared using the chi‐square test. Possible predictors of clinical outcomes were first investigated using univariate analysis. These were then subjected to multivariate logistic regression analysis to identify independent predictors and obtain odds ratio (OR) with 95% confidence interval (CI). SPSS version 18 software package (SPSS Inc., Chicago, Illinois) was used for statistical analysis. Two‐sided *P* values of <0.05 were considered as indicative of statistical significance.

### Propensity score matching

In order to address potential biases in patient selection and the impact of differences in confounding baseline variables between the two groups, a 1:1 propensity score matching without replacement was performed. Propensity scores were calculated using a logistic regression model including the following normalized covariates: age, sex, age‐adjusted Charlson's co‐morbidity index, clinical frailty score, NYHA class, GWTG clinical risk score, NT‐proBNP, and left ventricular ejection fraction (EF). Mean and frequency imputations were used in case of covariate missing data. Patients within the control group were matched to patients from the interventional group (HFVW) using the ‘greedy nearest neighbour matching algorithm’ with a calliper value of 0.2 of the pooled standard deviation of the logs of the propensity score. Standardized mean differences (SMD) were calculated to compare baseline characteristics after matching. A post‐matching SMD < 0.1 was considered to denote negligible difference between the two groups. The common support assumption was assessed using the Kolmogorov–Smirnov nonparametric test. Common support intervals were determined using the trimming method and kernel density estimators with the threshold was set at 0.001.

## Results

### Baseline patient characteristics

In total 958 consecutive patients were included in this study (554 were in the HFVW cohort and 404 in the standard care cohort) and median follow‐up was for 364 days (range 90–613 days). *Table* *1* shows the baseline patient characteristics before and after propensity score matching. Prior to propensity score matching, the standard care cohort showed a trend towards higher clinical frailty score and consisted of a higher proportion of patients treated with intravenous diuretics although these were statistically not significant. Following propensity score matching, the two cohorts (*n* = 400 in each cohort) were more evenly matched in terms of baseline characteristics such as age, gender, co‐morbidities, clinical frailty score, GWTG‐HF score, baseline NT‐proBNP, renal function, and ejection fraction.

**Table 1:** Baseline patient clinical characteristics (pre‐ and post‐propensity score matching)

Patient characteristics	Pre‐propensity score matching			Post‐propensity score matching		
	Standard care (*n* = 404)	HFVW (*n* = 554)	*P*	Standard care (*n* = 400)	HFVW (*n* = 400)	*P* (SMD)
Age (years)	74.2 ± 11.8	73.1 ± 10.9	0.15	74.1 ± 10.4	73.6 ± 11.3	*P* = 0.76 (0.05)
Women	49%	46%	0.36	47%	46.1%	*P* = 0.72 (0.03)
Hypertension	73%	69%	0.33	70%	69%	0.76
Diabetes	36%	31%	0.11	36%	35%	0.78
Chronic kidney disease	55%	52%	0.31	55%	53.5%	0.67
Valve disease	24%	21%	0.51	23.5%	23%	0.87
IHD	33%	36%	0.36	33%	34%	0.76
Atrial fibrillation	40%	38%	0.55	40%	39%	0.79
NT‐proBNP (nG/L)	3313 (range 590–35 000)	3100 (range 427–35 000)	0.4	3210	3170	*P* = 0.6 (0.04)
eGFR (mL/min/1.73 m^2^)	48.7 ± 8.1	49.5 ± 9	0.12	48.9 ± 8	49.1 ± 8.1	0.4 (0.04)
LVEF	42.1 ± 9.1	43 ± 10	0.15	42.4 ± 9.1	42.6 ± 9.9	*P* = 0.9 (0.02)
Charlson co‐morbidity index	7.4 ± 2.6	7.1 ± 2.8	0.1	7.15 ± 2.5	7.05 ± 2.6	*P* = 0.3 (0.05)
Clinical frailty score	5.4 ± 1.8	5.2 ± 1.5	0.06	5.25 ± 1.3	5.2 ± 1.2	*P* = 0.4 (0.01)
GWTG	43.9 ± 6	43.3 ± 5	0.11	43.6 ± 6	43.3 ± 6	*P* = 0.8 (0.02)
Length of stay (days)	11.1 ± 5	10.5 ± 4.8	0.07	‐	‐	‐
IV furosemide	74%	68%	0.08	69%	68%	*P* = 0.5
Beta‐blockers	94%	97%	0.07	‐	‐	‐
ACEi/ARB/ARNI	91%	94%	0.23	‐	‐	‐
MRA	62%	67%	0.1	‐	‐	‐
SGLT2i	40%	48%	0.06	‐	‐	‐
				‐	‐	‐
Permanent pacemaker	5%	8%	0.7			
CRTp/D	10%	13%	0.14			
Cardiac transplant referrals	0	3 (1 patient underwent cardiac transplant)		‐	‐	‐
Advance care planning discussions and palliative care referrals	17%	22%	0.07	‐	‐	‐

A standardized mean difference (SMD) <0.1 indicates a lack of major imbalance. NB matching was not performed for HF therapies other than IV frusemide on the premise that being on a HF specialist VW (‘intervention cohort’) enables earlier initiation and optimization of these therapies.

The use of HF therapies was similar between the two cohorts though there was a trend towards higher use of beta‐blocker, SGLT2i, cardiac transplant referrals, and advance care planning discussions in the HFVW cohort (statistically not significant).

### Outcomes

#### Re‐hospitalizations

Multivariate logistic regression analysis (*Table* *2*) showed that management in HFVW predicted significantly lower odds of re‐hospitalization (all‐cause) throughout the follow‐up period (1, 3, 6, and 12 months), whereas increased GWTG‐HF and CFS predicted higher odds of re‐hospitalization for patients in both cohorts.

**Table 2:** Results of multivariate logistic regression analysis for predictors of clinical outcomes in HFVW

All patients	Re‐hospitalizations: odds ratio (95% CI)				Mortality: odds ratio (95% CI)			
	1 month	3 months	6 months	12 months	1 month	3 months	6 months	12 months
Age	1.02 (0.93–1.05) *P* = 0.6	0.99 (0.96–1.03) *P* = 0.6	0.95 (0.93–1.04) *P* = 0.15	1.01 (0.95–1.05) *P* = 0.4	0.99 [0.95–1.02) *P* = 0.5	1.03 (0.97–1.08) *P* = 0.4	0.98 (0.94–1.02) *P* = 0.3	1.02 (0.96–1.04) *P* = 0.3
Sex	1.12 (0.7–1.9) *P* = 0.5	1.5 (0.8–2.8) *P* = 0.2	1.4 (0.8–2.4) *P* = 0.2	1.3 (0.5–3) *P* = 0.6	1.05 (0.6–1.9) *P* = 0.9	2.2 (0.9–5.2) *P* = 0.1	2.02 (0.8–4.6) *P* = 0.101	0.8 (0.2–5) *P* = 0.8
CCI	1.02 (0.93–1.13) *P* = 0.6	0.93 (0.82–1.06) *P* = 0.3	0.99 (0.88–1.12) *P* = 0.9	1.2 (1.1–1.4) *P* = 0.03	0.99 (0.88–1.12) *P* = 0.9	1.14 (0.96–1.34) *P* = 0.1	1.05 (0.89–1.25) *P* = 0.55	1.2 (1.1–1.4) *P* = 0.04
CFS	1.9 (1.5–2.4) *P* < 0.0001	1.8 (1.3–2.4), *P* = 0.0003	1.4 (1.1–1.8) *P* = 0.015	1.9 (1.2–3) *P* = 0.01	2.1 (1.6–2.8) *P* < 0.0001	2.34 (1.5–3.6) *P* = 0.0001	3.9 (1.2–7) *P* = 0.03	2.6 (1.6–7) *P* = 0.02
NYHA class	0.5 (0.2–1.1) *P* = 0.08	1.8 (0.8–4.3) *P* = 0.2	1.4 (0.9–3) *P* = 0.25	1.3 (0.7–2.5) *P* = 0.4	0.3 (0.1–1.7) *P* = 0.2	0.74 (0.2–2.4) *P* = 0.6	1.3 (0.9–3) *P* = 0.6	1.4 (0.4–3.8) *P* = 0.5
GWTG	1.2 (1.1–1.3) *P* < 0.001	1.5 (1.4–1.64) *P* < 0.001	1.3 (1.2–1.4) *P* < 0.001	1.1 (1.05–1.2) *P* = 0.03	1.2 (1.1–1.3) *P* < 0.001	1.3 (1.2–1.4) *P* < 0.001	1.2 (1.1–1.3) *P* < 0.001	1.3 (1.1–1.7) *P* = 0.02
NT‐proBNP	1.01 (0.99–1.02) *P* = 0.5	1 (0.99–1.01) *P* = 0.1	1.05 (0.85‐ 1.2) *P* = 0.3	1.01 (0.99–1.02) *P* = 0.25	1 (0.99–1.01) *P* = 0.2	1 (0.99–1.01) *P* = 0.1	1 (0.98–1.01) *P* = 0.15	1.02 (0.96–1.04) *P* = 0.3
EF	1.0, (0.97–1.02) *P* = 0.76	1.02 (0.99–1.1) *P* = 0.1	1.02, (0.99–1.05) *P* = 0.1	1.2 (0.3–5) *P* = 0.8	0.98 (0.95–1.01) *P* = 0.2	1.01 (0.97–1.05) *P* = 0.61	1.02 (0.98–1.06) *P* = 0.3	0.5 (0.3–2.5) *P* = 0.9
HFVW management	0.3 (0.2–0.5) *P* < 0.0001	0.15 (0.1–0.3) *P* < 0.0001	0.35 (0.2–0.6) *P* = 0.0002	0.25 (0.15–0.4) *P* < 0.001	0.3 (0.1–0.5) *P* < 0.0001	0.11, (0.04–0.3) *P* < 0.0001	0.35 (0.2–0.6) *P* = 0.0002	0.6 (0.5–0.7) *P* = 0.03
%Change step count	0.85 (0.7–0.9) *P* = 0.005	0.95 (0.93–0.98) *P* = 0.003	1.1 (0.85–1.2) *P* = 0.2	1.05 (0.9–1.1) *P* = 0.3	0.85 (0.7–0.95) *P* = 0.01	0.99 (0.98–1) *P* = 0.3	1.1 (0.9.‐ 1.2) *P* = 0.25	1.05 (0.85–1.1) *P* = 0.33

**Figure 3:**
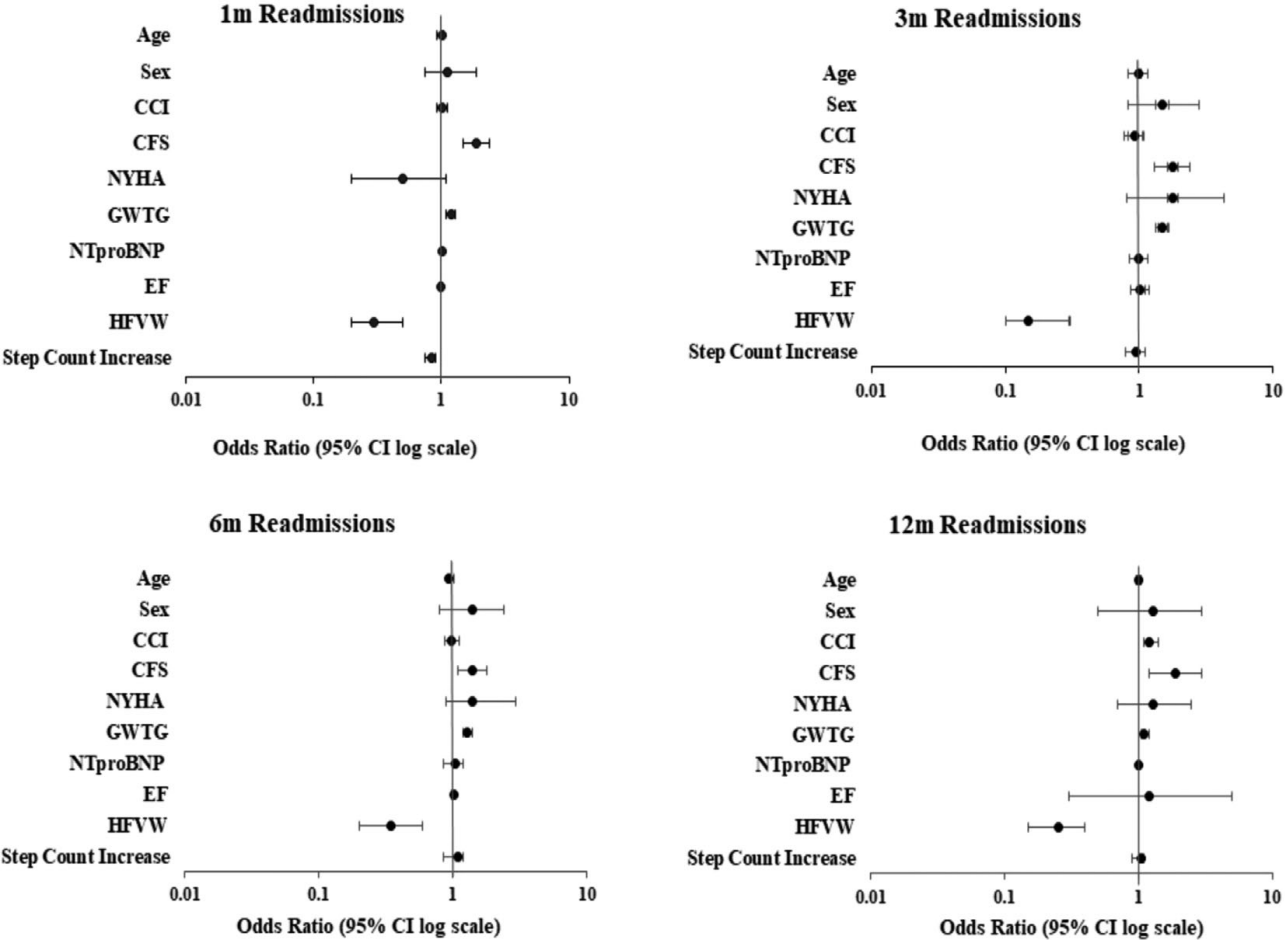
Multivariate logistic regression analysis with odds ratio for re‐admissions. CI, confidence interval.

Multivariate analysis (Table 2 and Forest plot in Figure 3) showed that compared with standard care, HFVW care was associated with lower odds of readmission (1‐month OR = 0.3 [95% confidence interval CI 0.2–0.5], *P* < 0.0001; 3 month OR = 0.15 [95% CI 0.1–0.3], *P* < 0.0001; 6‐month OR = 0.35 [95% CI 0.2–0.6], *P* = 0.0002; 12‐month OR = 0.25 [95% CI 0.15–0.4], *P* ≤ 0.001. Higher GWTG‐HF score independently predicted increased odds of re‐hospitalization (1‐month OR = 1.2 [95% CI 1.1–1.3], *P* < 0.001; 3‐month OR = 1.5 [95% CI 1.37–1.64], *P* < 0.0001; 6‐month OR = 1.3 95% CI [1.2–1.4], *P* < 0.0001; 12‐month OR 1.1 [95% CI 1.05–1.2], *P* = 0.03). Similarly higher CFS also independently predicted increased odds of re‐hospitalizations (1‐month OR = 1.9, [95% CI 1.5–2.4], *P* < 0.0001; 3‐month OR = 1.8 [95% CI 1.3–2.4], *P* = 0.0003; 6‐month OR = 1.4 [95% CI 1.1–1.8], *P* = 0.015; 12‐month OR 1.9 [95% CI 1.2–3], *P* = 0.01. Increased daily step count while on HFVW independently predicted reduced odds of re‐hospitalizations (1‐month OR = 0.85 [95% CI 0.7–0.9], *P* = 0.005; 3‐month OR = 0.95 [95% CI 0.93–0.98], *P* = 0.003) but not beyond this period. Higher CCI predicted greater odds of 12‐month re‐hospitalizations [OR 1.2 (95% CI 1.1–1.4), *P* = 0.03]. Other co‐variates, such as age, sex, NYHA class on admission, NT‐proBNP, and EF, were not associated with risk of re‐hospitalizations.

**Figure 4:**
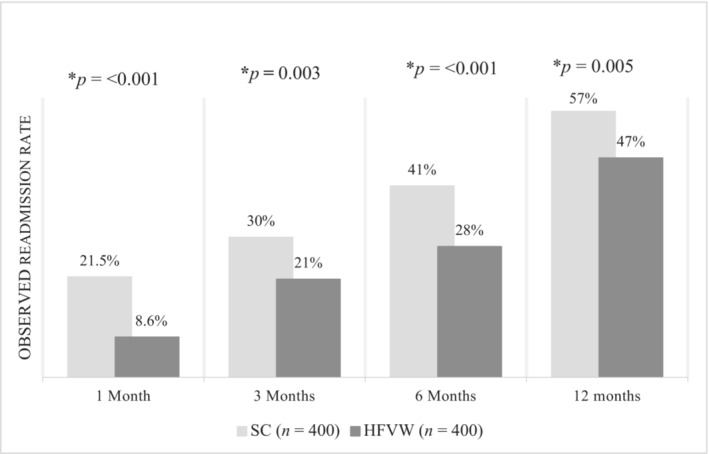
This graph outlines the observed re‐hospitalization rates in the two cohorts (SC, standard care and HFVW, heart failure virtual ward) at 1, 3, 6, and 12 months follow up. Significance is shown with *P*‐values and an asterisk (*) where appropriate.

As shown in *Table* *3* and *Figure* *4*, re‐hospitalizations were significantly lower when assessed after propensity score matching in the HFVW cohort compared with the matched standard care cohort throughout the follow‐up period (1 month re‐hospitalizations HFVW 8.6% vs. standard care 21.5%, *P* < 0.001; 3 months HFVW 21% vs. standard care 30%, *P* = 0.003; 6 months HFVW 28% vs standard care 41%, *P* < 0.001 and 12 months HFVW 47% vs. standard care 57%, *P* = 0.005).

**Table 3:** Patient outcomes

	Pre‐PSM			Post PSM		
Outcome	Standard care (*n* = 402)	HFVW (*n* = 554)	*P*	Standard care (*n* = 400)	HFVW (*n* = 400)	*P*
1‐month all‐cause re‐hospitalizations	21%	11.6%	0.002	21.5%	8.6%	<0.001
1‐month mortality	14%	6%	<0.001	13.7%	5%	<0.001
3‐month all‐cause re‐hospitalizations	30%	20.4%	0.001	30%	21%	0.003
3‐month mortality	15%	10.5%	0.02	15%	9.5%	0.001
6‐month re‐hospitalizations	41%	29.3%	0.02	41%	28%	<0.001
6‐month mortality	21%	15.5%	0.04	21%	15%	0.03
1‐year re‐hospitalizations	57%	48%	0.03	57%	47%	0.005
1‐year mortality	26%	21%	0.1	26%	20%	0.04
Hospital acquired infections	19%	4%	<0.001	‐	‐	‐
Falls	9%	2%	<0.001	‐	‐	‐
Adverse drug reactions	21%	5.5%	<0.01	‐	‐	‐

Throughout the follow‐up period, most of the re‐admissions to hospital were due to ‘non‐HF’ causes in both cohorts of patients (HFVW cohort 67% vs. standard care 65%). Multivariate analysis showed that GWTG‐HF score was the only independent predictor of HF re‐hospitalization (OR = 1.2, 95% CI [1.1–1.4], *P* = 0.03).

#### Mortality

**Figure 5:**
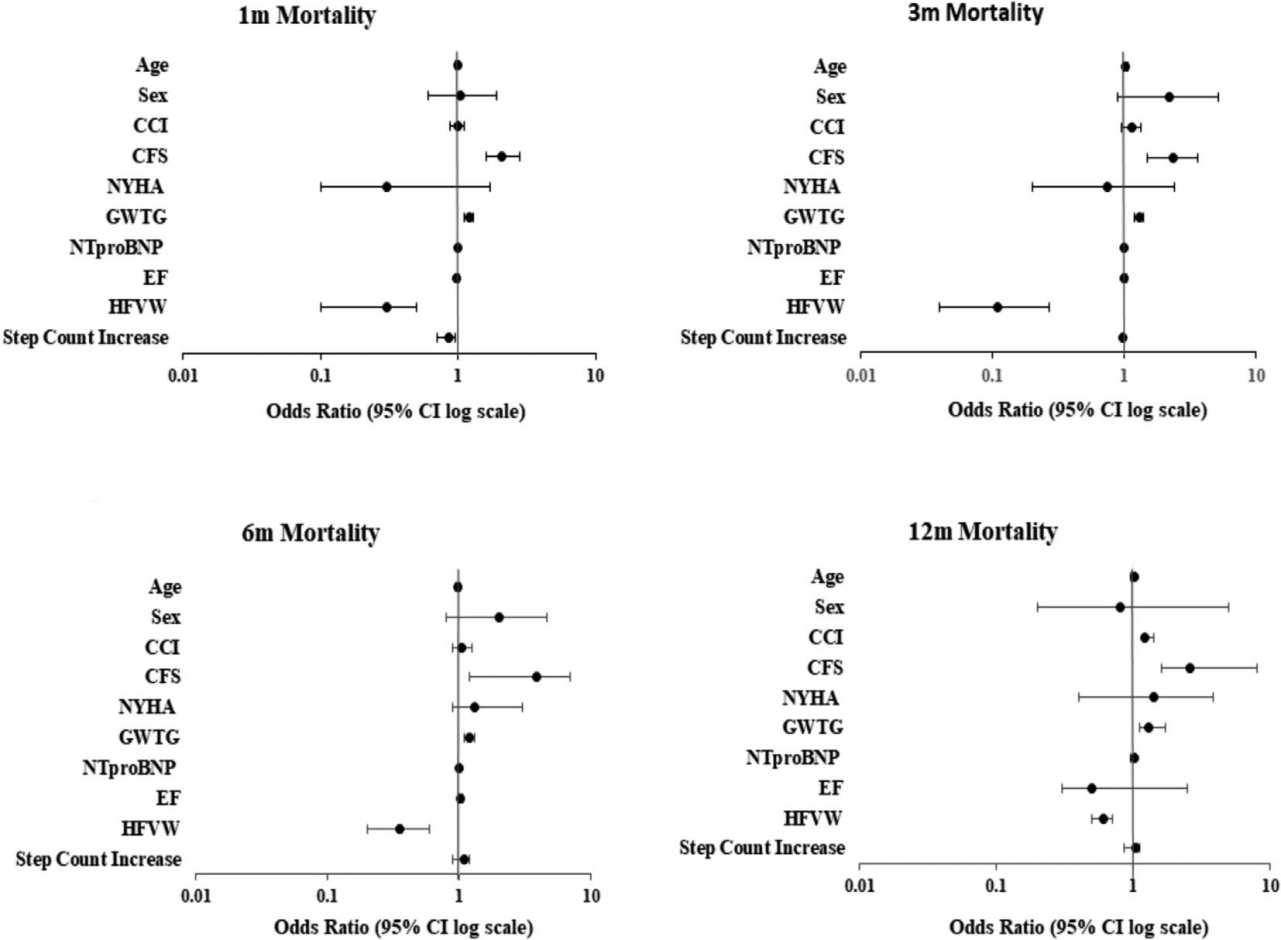
Multivariate logistic regression analysis with odds ratio for mortality. CI, confidence interval.

As shown in *Table* *2* and *Figure* *5*, multivariate logistic regression analysis showed that when compared with standard care, HFVW care independently predicted lower odds of mortality throughout the follow‐up period (1‐month OR = 0.26 [95% CI 0.14–0.48], *P* < 0.0001; 3‐month OR = 0.11 [95% CI 0.04–0.27], *P* < 0.0001; 6‐month OR = 0.35 [95% CI 0.2; 0.61], *P* = 0.0002; 12‐month OR = 0.6 [95% CI 0.48; 0.73], *P* = 0.03. *Table* *2* details multivariate logistic regression analysis which assessed other covariates for association with increased odds of mortality, and these are also illustrated in the Forest plots in *Figure* *5*.

Higher GWTG‐HF score independently predicted increased odds of mortality across both cohorts (1‐month OR = 1.21 [95% CI 1.1–1.3], *P* < 0.0001; 3‐month OR = 1.3 [95% CI 1.2–1.4], *P* < 0.0001; 6‐month OR = 1.2 [95% CI 1.1–1.3], *P* < 0.0001; 12‐month OR 1.3 [95% CI 1.1–1.7], *P* = 0.02). Higher CFS independently predicted mortality across both cohorts (1‐month OR = 2.1 [95% CI 1.6–2.8], *P* < 0.0001; 3‐month OR = 1.8 [95% CI 1.2–2.6], *P* = 0.006; 6‐month OR = 2.34 [95% CI 1.51–5.6], *P* = 0.0001; 12‐month OR 2.6 [95% CI 1.6–7], *P* = 0.02) throughout the follow‐up period. Increased daily step count while on HFVW independently predicted reduced odds of 1‐month mortality (OR 0.85 [95% CI 0.7–0.95], *P* = 0.01) but not beyond this period. A higher burden of co‐morbidities as assessed by CCI predicted greater odds of 12‐month mortality (OR 1.2; 95% CI [1.1 to 1.4], *P* = 0.04). Other co‐variates, such as age, sex, NYHA class on admission, NT‐proBNP, and EF, were not associated with increased risk of mortality.

**Figure 6:**
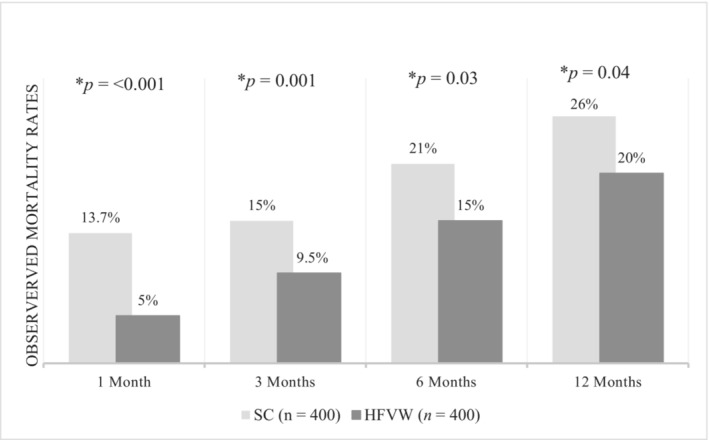
This graph outlines the observed mortality rates in the two cohorts (SC, standard care and HFVW, heart failure virtual ward) at 1, 3, 6, and 12 months follow up. Significance is shown with *P*‐values and an asterisk (*) where appropriate.

The incidence of mortality (Figure 6) was also significantly lower in the propensity score matched HFVW cohort in comparison to the standard care cohort throughout the follow‐up period (1‐month mortality HFVW 5% vs. SC 13.7%, *P* < 0.001; 3‐month mortality HFVW 9.5% vs. SC 15%; *P* = 0.001; 6‐month mortality HFVW 15% vs. SC 21%, *P* = 0.03; 12‐month mortality HFVW 20% vs SC 26%, *P* = 0.04).

## Discussion

To our knowledge, this is the first report describing the outcomes of patients with acute HF (new diagnosis as well as acute decompensation of previously diagnosed HF) managed with telehealth support in a HF specialist VW and in our propensity score matched analysis, this model of care has shown a reduction in all‐cause hospitalizations as well as mortality. In our study, the HFVW cohort also experienced a reduction in hospital acquired adverse outcomes such as hospital‐acquired infections, adverse drug reactions, and falls. Our results have demonstrated that management of acute decompensated HF in a specialist HFVW aided by telehealth may offer a safe as well as efficacious alternative to hospitalization in suitable patients. The above beneficial outcomes measures were seen in a HFVW cohort of patients with ADHF, compared with a hospitalized HF cohort, with both cohorts propensity score matched for potential confounders such as baseline patient characteristics (age, comorbidities, frailty, and clinical risk scores). It is important to note that the oldest patient managed in our HFVW was 97 years old, and hence, patient characteristics such as age, frailty, and cognitive impairment should not on their own preclude from management on the HFVW. Instead, care should be personalized towards assessment and management of each individual patient, who may also have carer support to help provide telehealth readings. Older or frailer patients may also require a more blended approach (with a combination of remote monitoring and face‐to‐face reviews).

Our study results have shown that GWTG‐HF clinical risk score and Rockwood CFS are independent predictors of short‐term as well as longer‐term adverse clinical outcomes such as re‐hospitalization and mortality, in both hospitalized as well as HFVW patients. While the GWTG‐HF score has been validated for the prediction of inpatient mortality amongst patients hospitalized with ADHF^22^ and there is some evidence suggesting longer term risk‐predictive utility,^23^ the predictive value of GWTG‐HF has not been assessed previously for out‐patient management of ADHF. Similarly, the prognostic value of CFS in the outpatient management of acute decompensated HF has not been studied before although evidence does suggest its utility as a risk marker.^24^, ^25^ Therefore, it is important to consider incorporating GWTG and CFS into routine clinical assessment at the time of first presentation with ADHF and also utilize these to assess risk and suitability of in‐patient versus out‐patient management of ADHF. Co‐morbidities as assessed using the CCI were shown in our study to predict 1 year outcomes (re‐hospitalizations and mortality), in line with previous evidence.^25^, ^26^ Our study has also offered interesting insights regarding the use of wearables to assess an increase in step count as a marker of improved physical activity in a HFVW population. Our results show that patients with a reduction in step count or a blunted increase in step count at the time of discharge from the HFVW have a higher risk of adverse outcomes up to 3 months following discharge. The use of physical activity and step counts to predict morbidity and mortality has been shown to be useful in other studies but not in an acute heart failure HFVW cohort previously.^27^ Assessment of daily step count using wearables should be considered as a risk predictor for adverse outcomes in the short‐term and lack of increase or a decrease in step count should prompt action such as assessment of volume status, need for early optimization of prognostic therapies, reassessment of biomarkers, and prompt referral for advanced heart failure therapies or advance care planning discussions as appropriate.

Previous studies^28^, ^29^, ^30^ of multidisciplinary heart failure specialist care when continued following discharge from hospital with ADHF have shown a significant reduction (ranging from 35% to 50%) in 6‐month re‐hospitalization for HF. Incorporation of telehealth to these post‐discharge strategies has shown mixed results in randomized controlled trials (RCTs) in terms of impact on clinical outcomes such as re‐admissions, mortality, and quality of life. Some RCTs showed a lack of significant benefit^31^, ^32^, ^33^ whereas others^34^ showed a reduction in percentage of days lost due to unplanned cardiovascular hospitalization or all‐cause death. A meta‐analysis of nearly 11 000 patients from 29 trials showed that post‐discharge telemonitoring can help reduced HF hospitalizations and improve quality of life.^35^ More recently, there has also been a greater focus on outpatient intravenous diuretic therapy instead of hospitalization, and this strategy has been shown to be safe and efficacious.^36^, ^37^, ^38^ Our HFVW strategy has combined the use of telehealth in the ambulatory or outpatient management of ADHF, and the improved clinical outcomes suggest that this can be a viable management option amongst suitable patients.

The predictors of adverse clinical outcomes due to HF decompensation (poor quality of life, re‐hospitalization, and mortality) are multifactorial. These include patient age, NYHA class, clinical parameters at the time of initial presentation (systolic blood pressure, heart rate, and oxygen saturation), abnormal serum biomarkers, co‐morbidities, and frailty.^39^, ^40^, ^41^ The two cohorts studied in our project (HFVW vs. hospitalized HF patients) did not show statistically significant differences in these parameters; however, to further minimize any potential confounding effect or bias, we ensured that comparisons were performed after propensity score matching.

Our model of management of acute decompensated HF in a heart failure specialist HFVW aided by remote monitoring analysed by a dedicated telehealth centre, attempts to simulate hospital management of ADHF. This includes daily detailed cardiac and HF symptom questionnaire, administration of intravenous diuretics, point of care testing, checking clinical parameters thrice daily, daily weights, daily ECG but also a wearable step count. The TIMHF‐2 study showed similarly that multi‐parametric data obtained by a dedicated telemedicine centre, contributed to improved effectiveness of remote HF management and thereby led to improved patient outcomes.^34^

Other factors that are also known to influence outcomes in hospitalized HF patients include their place of care^42^ (cardiology ward versus non‐cardiology ward) and management as well as follow‐up by HF specialists. As seen in the HF HFVW cohort, management by HF specialists allows for earlier initiation as well as better optimization of prognostic HF therapies, early referral for advanced HF therapies and also advance care planning discussions to enable timely palliative care, avoid unnecessary hospitalizations and facilitate home‐based care strategies in end‐stage HF. Diversion of suitable ADHF patients from A&E setting to a HF specialist HFVW such as in our model enables early management to be maintained by the HF specialist team. Patients from our HF HFVW also benefit from timely discussion with other specialists in our regional multi‐speciality MDT meeting for optimal management of their multi‐morbidity and this in turn has shown a reduction in all‐cause re‐hospitalizations.^18^ Another factor that possibly contributed to improved outcomes in the HFVW cohort was the early access to the Aintree HF Passport Mobile App, which has been shown to lead to improved HF self‐care behaviour, better medication adherence and also helps reduce 30‐day re‐hospitalizations.^43^

Hospitalization due to HF has been shown to independently predict mortality and portend poor quality of life. In addition, hospitalization also leads to disorders in physiological homeostasis such as sleep deprivation and disturbed circadian rhythm, poor nutrition, deconditioning due to a lack of physical activity during hospitalization, pain or discomfort, cognitive impairment, and delirium.^44^, ^45^ These combine to contribute to a distinctive syndrome called ‘post‐hospital syndrome’, which is a period during which there is enhanced vulnerability, impaired recovery and susceptibility to further re‐admission. Iatrogenic complications have been shown to occur in 26% of HF hospitalizations and previous unpleasant experiences with hospital care can also lead to a delay in seeking help.^46^, ^47^ In contrast, a home‐ based disease management programme can lead to improved mental well‐being (reduced anxiety and depression).^48^ It is likely that these factors along with the reduction in hospital acquired infections, adverse drug reactions (including due to prescribing errors on non‐specialist wards or by non‐specialists during evenings or weekends), and falls (generally due to hospital acquired delirium) led to improved outcomes amongst the HFVW cohort.

## Limitations

We acknowledge that our results are from an observational study, with limitations known to affect non‐randomized studies and therefore our conclusions should be considered as hypothesis generating. However, we have endeavoured to alleviate the impact of any potential confounding bias due to differences in covariates by subjecting the two cohorts to propensity score matching and demonstrated the real‐world effectiveness and safety of virtual ward management which has been implemented widely across the UK health service. The HFVW model will also need to be subjected to a health economic analysis to ascertain cost‐effectiveness.

## Conclusion

Our propensity score matched observational study of the remote monitoring telehealth guided out‐patient management of ADHF by a HF specialist team suggests that in suitable patients, this can be a safe strategy but also lead to improved clinical outcomes such as reduced re‐hospitalizations and mortality. Consideration should be given to incorporate assessment of GWTG‐HF clinical risk score, clinical frailty score, Charlson's co‐morbidity index as a part of clinical assessment during presentation with ADHF as these can help predict adverse outcomes and also help guide decision‐making regarding hospitalization versus out‐patient management. Measurement of daily step count using wearables during management of ADHF can offer valuable insights regarding patients at early risk of adverse outcomes such as re‐hospitalization and mortality.

## Conflict of interest

None relevant to this study.

## Funding

The Liverpool Acute Heart Failure Virtual Ward was funded initially as a pilot study by a Digital Transformation Award from NHSX (NHS Transformation Directorate) with £237 000. For the purpose of Open Access, the author has applied a Creative Commons Attribution (CC‐BY) licence to any Author Accepted Manuscript version arising.

## Supporting information


**Data S1.** Supporting Information.
