# Supplementary material for: Telehealth‐aided outpatient management of acute heart failure in a specialist virtual ward compared with standard care
Source: ESC Heart Fail. 2024 Aug 13;11(6):4172–84. doi: 10.1002/ehf2.15003 (PMC11631251; doi:10.1002/ehf2.15003)
Supplement: Supplementary file 1 — Data S1. Supporting Information. [file EHF2-11-4172-s001.docx]

**Supplementary Appendix**

GWTG-HF Score

| GWTG-HF | Systolic blood pressure (mmHg) | |
| --- | --- | --- |
|  | ≥200 | 0 |
|  | 190-199 | 2 |
|  | 180-189 | 4 |
|  | 170-179 | 6 |
|  | 160-169 | 8 |
|  | 150-159 | 9 |
|  | 140-149 | 11 |
|  | 130-139 | 13 |
|  | 120-129 | 15 |
|  | 110-119 | 17 |
|  | 100-109 | 19 |
|  | 90-99 | 21 |
|  | 80-89 | 23 |
|  | 70-79 | 24 |
|  | 60-69 | 26 |
|  | 50-59 | 28 |
|  | BUN | |
|  | ≤9 | 0 |
|  | 10-19 | 2 |
|  | 20-29 | 4 |
|  | 30-39 | 6 |
|  | 40-49 | 8 |
|  | 50-59 | 9 |
|  | 60-69 | 11 |
|  | 70-79 | 13 |
|  | 80-89 | 15 |
|  | 90-99 | 17 |
|  | 100-109 | 19 |
|  | 110-119 | 21 |
|  | 120-129 | 23 |
|  | 130-139 | 25 |
|  | 140-149 | 27 |
|  | ≥150 | 28 |
|  | Sodium | |
|  | ≥139 | 0 |
|  | 137-138 | 1 |
|  | 134-136 | 2 |
|  | 131-133 | 3 |
|  | ≤130 | 4 |
|  | Age | |
|  | ≤19 | 0 |
|  | 20-29 | 3 |
|  | 30-39 | 6 |
|  | 40-49 | 8 |
|  | 50-59 | 11 |
|  | 60-69 | 14 |
|  | 70-79 | 17 |
|  | 80-89 | 19 |
|  | 90-99 | 22 |
|  | 100-109 | 25 |
|  | ≥110 | 28 |
|  | Heart rate | |
|  | ≤79 | 0 |
|  | 80-84 | 1 |
|  | 85-89 | 3 |
|  | 90-94 | 4 |
|  | 95-99 | 5 |
|  | 100-104 | 6 |
|  | ≥105 | 8 |
|  | COPD | |
|  | No | 0 |
|  | Yes | 2 |
|  | Black race | |
|  | No | 3 |
|  | Yes | 0 |
|  | Total GWTG score = Systolic BP score + BUN score + sodium score + age score + heart rate score + COPD score + Black race | Range: 0-101 |
